# Conjugation of CRAMP_18–35_ Peptide to Chitosan and Hydroxypropyl Chitosan via Copper-Catalyzed Azide–Alkyne Cycloaddition and Investigation of Antibacterial Activity

**DOI:** 10.3390/ijms25179440

**Published:** 2024-08-30

**Authors:** Sankar Rathinam, Kasper K. Sørensen, Martha Á. Hjálmarsdóttir, Mikkel B. Thygesen, Már Másson

**Affiliations:** 1 Faculty of Pharmaceutical Sciences, School of Health Sciences, University of Iceland, Hofsvallagata 53, IS-107 Reykjavík, Iceland; sankar@hi.is; 2 Department of Chemistry, Faculty of Science, University of Copenhagen, Thorvaldsensvej 40, DK-1871 Frederiksberg C, Denmark; kso@chem.ku.dk (K.K.S.); mbth@chem.ku.dk (M.B.T.); 3 Department of Biomedical Science, Faculty of Medicine, School of Health Sciences, University of Iceland, Hringbraut 31, IS-101 Reykjavík, Iceland; hjalmars@hi.is

**Keywords:** chitosan, click chemistry, antimicrobial peptide, antibacterial activity

## Abstract

We developed a synthesis strategy involving a diazo transfer reaction and subsequent click reaction to conjugate a murine cathelicidin-related antimicrobial peptide (CRAMP_18–35_) to chitosan and hydroxypropyl chitosan (HPC), confirmed the structure, and investigated the antimicrobial activity. Chitosan azide and HPC-azide were prepared with a low degree of azidation by reacting the parent chitosan and HPC with imidazole sulfonyl azide hydrochloride. CRAMP_18–35_ carrying an N-terminal pentynoyl group was successfully grafted onto chitosan and HPC via copper-catalyzed azide–alkyne cycloaddition (CuAAC) reaction. The chitosan–peptide conjugates were characterized by IR spectroscopy and proton NMR to confirm the conversion of the azide to 1,2,3-triazole and to determine the degree of substitution (DS). The DS of the chitosan and HPC CRAMP_18–35_ conjugates was 0.20 and 0.13, respectively. The antibacterial activity of chitosan–peptide conjugates was evaluated for activity against two species of Gram-positive bacteria, *Staphylococcus aureus* (*S. aureus*) and *Enterococcus faecalis* (*E. faecalis*), and two species of Gram-negative bacteria*, Escherichia coli* (*E. coli*) and *Pseudomonas aeruginosa* (*P. aeruginosa*). The antimicrobial peptide conjugates were selectively active against the Gram-negative bacteria and lacking activity against Gram-positive bacteria.

## 1. Introduction

Antimicrobial peptides (AMPs) are natural components of the innate immune system in both vertebrates and invertebrates, and they play a prominent role as effector molecules [1,2]. AMPs can be classified based on the source, such as mammals (human host defense peptides), amphibians, microorganisms, and insects [3]. Researchers have studied AMPs as a potential alternative to current antibiotic treatments or as an addition to existing therapies [4]. Antimicrobial peptides (AMPs) are highly effective in selectively inhibiting the growth of bacteria [5,6], fungi [7,8], and viruses [9]. As an alternative to conventional antibiotics, AMPs have the advantage of low resistance induction and antimicrobial effects [10]. These peptides are composed of amino acid residues ranging from 10 to 60 units. Most AMPs are cationic and structurally amphipathic, which makes them suitable for binding to the bacterial cell membrane of potential pathogens and permeabilizing the membrane [11].

Antimicrobial peptides display a considerable range of structures and have a broad antimicrobial spectrum. Mammalian antimicrobial peptides are present in humans, sheep, cattle, pigs, rabbits, and other vertebrates. Two AMP families are generally recognized: cysteine-rich defensins (α- and β-defensins) and various cathelicidins [12]. Cathelicidins originate from a preprotein containing an N-terminal domain called cathelin, a putative cysteine-protease inhibitor, and a C-terminal domain consisting of the antimicrobial peptide [13]. The C-terminal domains exhibit structural diversity, with α-helical, β-sheet, β-turn, or extended conformations [14]. The gene family of cathelicidin is identified from full-length cDNA sequences; human cathelicidin is named LL-37 and has a length of 37 amino acids, while its homologue in mice is named CRAMP (or mCRAMP), cathelicidin-related antimicrobial peptide, with a length of 38 amino acids [15]. CRAMP is a potent cationic antimicrobial peptide that effectively combats both Gram-negative and Gram-positive bacteria as well as human fungal pathogens while maintaining a low hemolytic activity [16,17]. CRAMP is believed to exert its antimicrobial effect primarily by membrane perturbation and permeabilization of pathogenic cell walls [15,18].

Previous studies demonstrated that the CRAMP sequence from amino acid units 16 to 33 [17,19] inhibits bacterial cytokinesis, and thus has a multifaceted antimicrobial activity profile [20]. Further studies found that the amino acid sequence from 18 to 35 (CRAMP_18–35_) maintains anti-biofilm activity [19,21], and a minimal sequence was later identified as amino acids 20 to 33 [22]. An AMP’s antimicrobial effectiveness and mechanism of action may vary due to factors such as concentration and hydrophobicity. Therefore, the relationship between the structure of AMP and its antimicrobial properties remains uncertain [23,24]. The development of AMPs immobilized on material surfaces via covalent coupling methods has also been realized. AMP-coated surfaces can inhibit a broad spectrum of microbes, thus preventing bacterial colonization and biofilm formation [25]. Different peptide conjugation strategies have been established, such as simple amide coupling reactions or introducing sulfur-reactive molecules to couple with cysteine-containing peptides and materials. However, some of these methods may be inappropriate for a stable bond under bio-reduction conditions [26]. To overcome these obstacles, chemo-selective methods such as click chemistry can be explored. The copper(I)-catalyzed azide–alkyne cycloaddition (CuAAC) reaction between terminal alkynes and azides has been elaborated in various areas of chemistry such as material sciences, drug discovery modification of DNA, natural products, nanoparticles, and bioconjugation to polymers [27,28,29,30].

One such polymer is chitosan, a linear polysaccharide composed of randomly distributed β-(1-4)-linked D-glucosamine and *N*-acetylated-D-glucosamine units. Chitosan and its derivatives constitute attractive biopolymers due to their biodegradability, biocompatibility, and low toxicity [31]. It gives beneficial physicochemical properties and biomedical applications [32]. Chitosan and its derivatives have shown enhanced antibacterial activity, whereas unmodified chitosan shows antibacterial activity under acidic conditions [33]. Chitosan derivatives were used as antimicrobials [34,35], for drug and gene delivery [36], as an absorption enhancer [37], and for tissue engineering [38]. We recently published a procedure to convert the *C*-2 amino groups into azides by diazo-transfer reaction and then to triazoles by CuAAC to obtain chitotriazolan derivatives that can have enhanced antibacterial activity [39,40]. Others have also reported using CuAAC to link chitosan cationic groups to the *C*-2 amino group and *C*-6 carbon [41,42]. Our group reported grafting anoplin antimicrobial peptides onto chitosan polymers through *N*-(2-azidoacetyl) groups using CuAAC [43]. The conjugates showed an enhanced antibacterial activity relative to the parent peptide and reduced non-hemolytic activity. Barbosa et al. [44] reported grafting antimicrobial 14-amino-acid peptide Dhvar-5 onto chitosan using diazo transfer and CuAAC, similar to our recently reported diazo transfer/CuAAC procedure. The peptide conjugation was confirmed by FTIR, XPS, and amino acid analysis, but the reported degree of substitution (DS) was low (DS ~ 0.008), and no NMR analysis was reported. Later, they reported that this conjugate lacked antibacterial activity in the solution but could be used in thin film coatings with bactericidal effects [44,45].

The objective of the current study was to assess the effectiveness of CRAMP_18–35_ peptides conjugated to chitosan polymers as antimicrobial agents. We hypothesized that our recently developed procedure for chitotriazolan synthesis would allow the conjugation at a higher DS than previously reported for Dhvar-5 conjugates, thus contributing to significant activity in the solution. For this purpose, the chitosan azide and hydroxypropyl chitosan (HPC) azide were prepared with a low degree of azidation, and subsequently, peptides were conjugated through the CuAAC reaction. These conjugates were characterized by IR, NMR spectroscopy, and SEC-MALS. The conjugates had better water solubility and were evaluated against four different bacteria, two Gram-positive bacteria and two Gram-negative bacteria.

## 2. Results and Discussion

The primary aim of the current investigation was to explore the possibility of enhancing the antimicrobial properties of chitosan and chitosan derivatives by incorporating a short antimicrobial peptide onto their backbone structure using our previously reported diazo transfer and CuAAC “click chemistry” procedure, in which aqueous DMSO is used as the solvent for chitosan and chitosan derivatives [46]. The chosen method for achieving this objective is the copper-catalyzed azide–alkyne cycloaddition reaction. This reaction involves coupling an azide-functionalized molecule (in this case, chitosan with azide groups) with an alkyne-functionalized molecule (the antimicrobial peptide) to form a triazole linkage. This methodology is selected based on previous findings and NMR investigations that indicated the successful conversion of chitosan’s *C*-2 amino groups to azide groups via a diazo transfer reaction, followed by a CuAAC reaction, resulting in the formation of triazole derivatives. These polymeric compounds, named chitotriazolans, display enhanced water solubility and antibacterial properties [39].

The rationale behind this approach is to take advantage of the unique properties of both chitosan and the antimicrobial peptide. Chitosan is known for its biocompatibility, biodegradability, and antimicrobial activity, making it an attractive material for various biomedical applications. By conjugating an antimicrobial peptide onto the chitosan backbone, we aim to combine the inherent antimicrobial properties of chitosan with the specific and potent antimicrobial activity of the peptide, potentially leading to selective antimicrobial effects. Chitosan is not readily soluble at pH 7.2, and the measured MIC values can be variable depending on the study. Our previous work found that the MIC for chitosan was 256 and 2048 µg/mL for activity against *S. aureus* and *E. coli*, respectively [35]. The same study found that hydroxypropylation improved solubility and reduced antimicrobial activity.

In the recent work published by our group, the synthesis strategy involved converting chitosan derivatives such as *N*,*N*,*N*-trimethyl chitosan (TMC), *N*-(2-(*N*,*N*,*N-*trimethylammoniumyl))acetyl chitosan (TAC), 2-hydroxy-3-(*N*,*N*,*N*-trimethylammoniumyl)propyl chitosan (HTC), *N*-(2-hydroxy)propyl chitosan (HPC), and *N*-carboxymethyl chitosan (CMC) to partially substituted derivatives, followed by conversion of the remaining primary amino groups to azides. This resulted in chitosan derivatives with triazole moieties, displaying improved water solubility and antibacterial activity [46]. We have designed the antimicrobial peptide conjugation onto chitosan based on our previous results and investigated the modified chitosan–peptide structure’s antimicrobial properties.

**Scheme 1:**
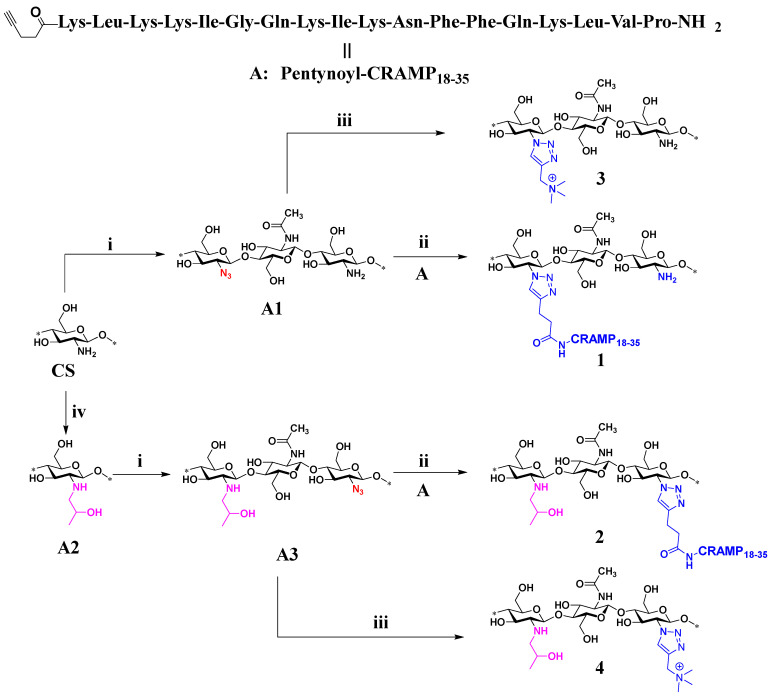
Conjugation of chitosan–CRAMP_18–35_ through diazo-transfer and subsequent click reaction; (**i**) 0.1 M HCl solution, sodium bicarbonate, imidazole sulfonyl azide HCl salt, CuSO_4_ 5H_2_O, water, methanol, RT; (**ii**) CuSO_4_ 5H_2_O, sodium ascorbate, pentynoyl–CRAMP_18–35_, DMSO, THPTA, 50 °C; (**iii**) CuSO_4_ 5H_2_O, sodium ascorbate, *N*-propargyl-*N,N,N*-trimethylammonium bromide, DMSO, 50 °C; (**iv**) propylene oxide, isopropyl alcohol, NaOH (30%). * asterisk bond for Polymer chain linkers continuation.

In the present work, the synthesis of biopolymers **1** and **2** was performed with a low degree of azidation of the chitosan and hydroxypropyl chitosan (**A2**) C-2 amino groups. Only 0.4 equivalents of imidazole sulfonyl azide HCl salt were used relative to the amino groups in chitosan. The CRAMP_18–35_ peptide sequence KLKKIGQKIKNFFQKLVP, was synthesized separately. The *N*-terminus CRAMP_18–35_ was then coupled with 4-pentynoic acid to obtain pentynoyl–CRAMP_18–35_, as illustrated in Scheme 1.

**Figure 1:**
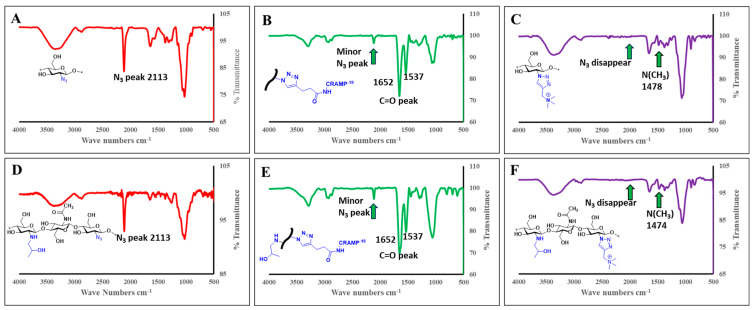
IR spectra of chitosan azide (**A1**) (**A**), CRAMP_18–35_-chitotriazolan (**1**) (**B**), chitotriazolan (**3**) (**C**), HPC azide (**A3**) (**D**), CRAMP_18–35_-HP-chitotriazolan (**2**) (**E**), and HP-chitotriazolan (**4**) (**F**). * asterisk bond for Polymer chain linkers continuation.

The chitosan azide intermediate was found to be insoluble in both aqueous and organic solvents, except for dimethyl sulfoxide (DMSO) at 50 °C. However, even in DMSO, the solubility was inadequate for proton nuclear magnetic resonance (NMR) analysis. This limitation necessitated the use of infrared (IR) spectroscopy as the primary analytical technique for characterizing the synthesized material. The CuAAC reaction between chitosan azide (**A1**) or hydroxypropyl chitosan azide (**A3**) and pentynoyl–CRAMP_18–35_ peptide was performed in the presence of tris(3-hydroxypropyltriazolylmethyl)amine (THPTA) as a ligand. THPTA accelerates the reaction and enhances the efficiency of bioconjugation by maintaining the copper(I) oxidation state while protecting the biomolecules from oxidative damage [47]. One equivalent of pentynoyl–CRAMP_18–35_ peptide was utilized in the reaction to produce CRAMP_18–35_ chitotriazolan (**1**) as depicted in Scheme 1. Despite the efficient reaction, some unreacted azide residue was retained in the product, as confirmed by IR spectroscopy (Figure 1B). This indicates that more pentynoyl–CRAMP_18–35_ peptide was required for full conversion. A similar procedure was followed for the conjugation of CRAMP_18–35_ onto hydroxypropyl chitosan (**2**), which also exhibited a residual amount of azido groups in the product (Figure 1E). To ensure complete conversion in subsequent reactions, a higher alkyne–azide ratio (1.5 to 2.0 equivalents) was employed to react with the chitosan azide. Using these conditions, parent structures of chitotriazolans, derivatives **3** and **4**, were synthesized in which the CuAAC reaction was performed with *N*-propargyl-*N,N,N*-trimethylammonium bromide (Scheme 1) to explore the impact of the antimicrobial peptide on antibacterial activity.

### 2.1. Characterization of Products by IR Spectroscopy

In this study, IR spectroscopy was employed to characterize the CRAMP_18–35_-grafted chitosan derivatives and other derivatives of chitosan. It provides insights into the functional group changes occurring during the synthesis process. The successful diazo transfer reaction for both chitosan azide (**A1**) and hydroxypropyl chitosan (HPC) azide (**A3**) is indicated by strong stretching vibrational frequencies observed at 2113 cm^−1^ (Figure 1A,D). This peak confirms the presence of azide groups in the respective derivatives. Following the CuAAC reaction to produce CRAMP_18–35_-grafted chitosan derivatives (Figure 1B,E), an enhanced sharp peak of carbonyl stretching frequency is observed at 1652 cm^−1^. This peak represents the merging of amide carbonyl (C=O) peaks from chitosan acetamides and peptide amido groups, indicating successful conjugation of CRAMP_18–35_ to the chitosan backbone. A peak at 1537 cm^−1^ corresponds to the aromatic phenyl (C=C) bending frequency, indicating the presence of phenyl groups in the structure. The azide peak at 2113 cm^−1^ is observed with a very low intensity, suggesting that the azide groups may not be fully converted to triazole. For the parent structures of chitotriazolan (**3**) and HP-chitotriazolan (**4**) (Figure 1C,F), a peak at 1475 cm^−1^ corresponds to the trimethylammonium (*N*-CH_3_) group, indicating the formation of triazole moieties in these derivatives. The IR spectra provide evidence of significant structural changes during the synthesis process, including the formation of triazole moieties, conjugation of CRAMP_18–35_ peptide to the chitosan backbone, and the presence of aromatic phenyl groups. These changes are crucial for understanding the functional properties and potential applications of synthesized derivatives.

### 2.2. Characterization of Products by NMR Spectroscopy

**Figure 2:**
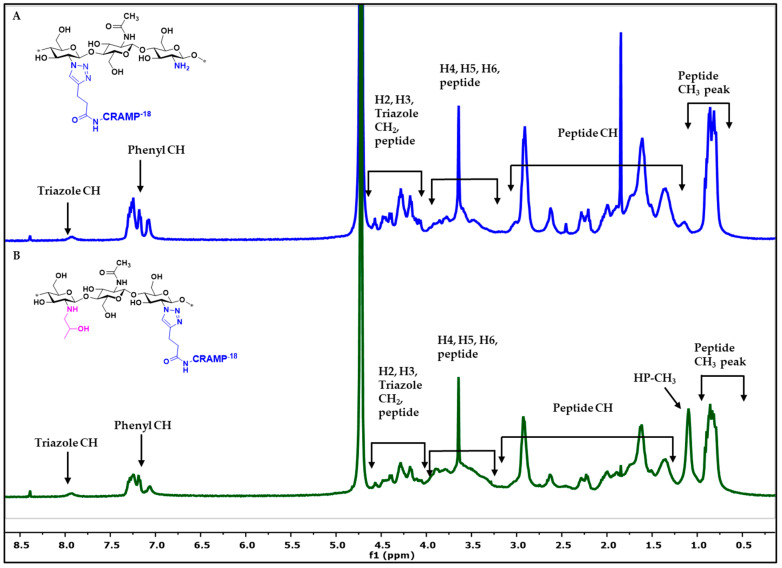
Proton NMR of CRAMP_18–35_ conjugated on CS **1** (**A**) and CRAMP_18–35_ conjugated on HPC **2** (**B**). * asterisk bond for Polymer chain linkers continuation.

The spectra for CRAMP_18–35_-grafted chitosan derivatives by NMR spectroscopy are shown in Figure 2. The presence of a triazole peak in the NMR spectrum confirms the successful conjugation of CRAMP_18–35_ to the chitosan backbone via the CuAAC reaction. This peak is observed at a chemical shift of 7.94 ppm. Another notable peak observed in the spectrum corresponds to the aromatic protons of phenylalanine, an amino acid present twice in the CRAMP_18–35_ peptide. This peak is identified in the range of 7.08 to 7.30 ppm (Figure 2A). The spectrum also reveals peaks corresponding to the aliphatic protons of CRAMP_18–35_ peptide molecules. These peaks are observed in the chemical shift range of 0.79 to 4.48 ppm, representing various aliphatic groups within the peptide sequence. In the NMR spectrum of CRAMP_18–35_-HP-chitotriazolan derivatives, an additional peak is observed corresponding to the hydroxypropyl methyl group. This peak appears at chemical shifts ranging from 1.09 to 1.13 ppm (Figure 2B). Overall, the NMR analysis provides valuable structural information regarding the CRAMP_18–35_-grafted chitosan derivatives, confirming the successful conjugation of CRAMP_18–35_ to the chitosan backbone and offering insights into the chemical environment within the resulting molecules. The degree of substitution (DS) was determined based on proton NMR spectra for the chitosan peptide conjugates and the other derivatives (Table 1). The DS for chitosan–CRAMP_18–35_ peptide (**1**) and hydroxypropyl chitosan (HPC)-CRAMP_18–35_ peptide (**2**) was determined to be 0.2 and 0.13, respectively. The molecular weight was determined by gel permeation chromatography, and the molecular weight of the chitosan peptide conjugates was increased compared to that of the unmodified chitosan (MW 108 kDa). A summary of the DS, DA, and MW data for conjugate **1**–**4** and chitosan derivative **A2** is reported in Table 1.

### 2.3. Antibacterial Analysis of Antimicrobial Peptide Chitosan Derivatives

This study evaluates the antibacterial properties of CRAMP_18–35_-grafted chitotriazolan derivatives using a serial dilution method. The antibacterial activity was assessed under physiological conditions (pH 7.2) against various bacterial strains. Specifically, Gram-positive bacteria included *S. aureus* and *E. faecalis*, while Gram-negative bacteria included *E. coli* and *P. aeruginosa*. The minimum inhibitory concentration (MIC) values obtained from this experiment are listed in Table 2. The CRAMP_18–35_-grafted chitotriazolan derivatives **1** and **2** exhibited activity against *E. coli* at concentrations of 256 µg/mL and against *P. aeruginosa* at concentrations of 1024 and 512 µg/mL, respectively. This indicates that these derivatives are effective against Gram-negative bacteria. However, their activity against Gram-positive bacteria was either absent or required high concentrations (≥4096 µg/mL), suggesting a selective antibacterial effect. We believe that the residual azide groups in derivatives **1** and **2** might not contribute significantly to their antibacterial activity. This inference is drawn from the observation that only a low degree of azide residue was retained in the antimicrobial peptide chitosan polymers [48]. The parent structures **3**, **4**, and **A2** displayed moderate activity against both Gram-positive and Gram-negative bacteria. In our previous work, we found that cationic chitotriazolan, with a structure similar to **3** but with a high degree of substitution, exhibited good activity [39]. However, in this instance, we prepared compound **3** with a low degree of substitution, leading to lower activity. The minimum lethal concentration (MLC), also referred to as the minimum bactericidal concentration (MBC), was determined. The measured MLC values were the same as the MIC values in all cases, indicating that the derivatives were bactericidal rather than bacteriostatic. On the other hand, structures **4** and **A2** demonstrated consistent activity levels, suggesting their potential as reliable antibacterial agents [35,46]. In summary, the study highlights the selective antibacterial activity of CRAMP_18–35_-grafted chitotriazolan derivatives against Gram-negative bacteria. Although several antimicrobial mechanisms of action have been suggested for chitosan, we have argued that the principal mechanism of action is disruption of the cell membrane, similar to the mechanism of action of antimicrobial peptides [49]. *S. aureus* is a Gram-positive bacterium with a single membrane surrounded by a thick peptidoglycan cell wall, whereas *E. coli* is a Gram-negative with a lipopolysaccharide-containing outer membrane that is separated from the inner cell membrane by a thin peptidoglycan cell wall layer. Our previous investigation showed that common chitosan derivatives are generally somewhat more active against *S. aureus* at pH 7.2 than against *E. coli* [35]. In the current study, we have observed a remarkable selectivity where the antimicrobial peptide conjugates were ten to twenty times more active against Gram-negative than Gram-positive bacteria. This is reminiscent of but more marked than the selectivity of the parent pentynoyl–CRAMP_18–35_ peptide. This may be caused by a selective interaction of the peptide moieties with lipopolysaccharides in the outer membrane. Future investigations may reveal the precise mechanism of action. Additionally, it underscores the importance of structural modifications in influencing the antibacterial efficacy of these compounds.

## 3. Materials and Methods

### 3.1. Material

Chitosan (S160302-1-2-3-4) was obtained from Primex ehf, Siglufjördur, Iceland. The average molecular weight was calculated (MW 108 kDa) from size exclusion chromatography data, and the degree of acetylation (DA) of 17% was calculated by using proton NMR spectroscopy. All reagent-grade chemicals were purchased from Merck, Sigma Aldrich (Darmstadt, Germany): Imidazole, sodium azide, sulfuryl chloride, trimethylamine solution, copper (II) sulfate pentahydrate, sodium ascorbate, hydrochloric acid, propargyl bromide, acetonitrile, formic acid, 4-pentynoic acid, and tris-hydroxypropyltriazolylmethylamine (THPTA ligand). *N^α^*-9-fluorenylmethoxycarbonyl (Fmoc) amino acids, *N,N*-dimethylformamide (DMF), *N*-methyl-pyrrolidone (NMP), *N*-[(1*H*-benzotriazol-1-yl)(dimethylamino)methylene]-*N*-methylmethanaminium hexafluorophosphate *N*-oxide (HBTU), 1-hydroxybenzotriazole (HOBt), trifluoroacetic acid (TFA), piperidine and *N,N*-diisopropylethylamine (DIPEA) were purchased from Iris Biotech (Marktredwitz, Germany). All solvents, including dimethyl sulfoxide (DMSO), *N,N*-dimethylformamide (DMF), dichloromethane (DCM), acetone, methanol, ethanol, and acetonitrile, were also obtained from Merck, Sigma Aldrich. De-ionized water was treated using a Milli-Q™ filtration system. Dialysis membranes (RC, Spectra/Por, MW cutoff 3500 Da, 45 mm) were purchased from Spectrum^®^ Laboratories Inc. (Rancho Dominguez, CA, USA) and Pur-A-Lyser Mega 3500 dialysis kit.

#### 3.1.1. Synthesis of Antimicrobial Peptide CRAMP_18–35_ (**A**)

The antimicrobial peptide was prepared by solid-phase peptide synthesis (SPPS) on a Biotage Initiator+ Alstra synthesizer by using the Fmoc strategy. A TentaGel S Rink Amide resin with 0.23 mmol/g loading capacity was used. The resin (0.2 mmol) was placed in a 30 mL syringe rector with a chemically resistant filter. The resin was swelled and washed with DCM and DMF. Then, Fmoc deprotection was carried out at room temperature by treatment with 40% piperidine in DMF (9 mL) for 3 min and followed by 20% piperidine in DMF (9 mL) for 10 min. The resin was washed with NMP (2 × 9 mL) and DCM (9 mL), then the resin was washed with DMF (9 mL) at 70 °C for 1 min. Peptide couplings were performed by treating the resin with Fmoc-protected amino acids (4 equiv.), HBTU (3.9 equiv.), HOBt (4 equiv.) dissolved in DMF, and DIPEA (7.4 equiv.) dissolved in NMP, then the reaction was heated to 75 °C for 10 min to ensure high incorporation of the amino acid. The coupling was then repeated. After the coupling, the resin was washed with NMP (2 × 9 mL). The deprotection and coupling steps were repeated till the final amino acid was attached. Finally, the pentynoic acid was coupled using pentynoic acid (4 equiv.), HBTU (3.9 equiv.), HOBt (4 equiv.) dissolved in DMF, and DIPEA (7.4 equiv.) dissolved in NMP. The resin was then washed with NMP (5 × 9 mL), then with DCM (6 × 9 mL). The peptide was then released from the resin, and side-chain protecting group removal was performed simultaneously by treatment with TFA:H_2_O (95:5) at room temperature for 2 h. The TFA filtrate was evaporated using nitrogen and the peptide was precipitated using diethyl ether, centrifugated, and the peptide collected. The crude CRAMP_18–35_ was then purified on a reversed-phase high-performance flash chromatography (RP-HPFC) Selekt system from Biotage, using a C18 column (Sfär Bio C18 50 g) and CH_3_CN/H_2_O with 0.1% formic acid as eluants. The elution consisted of a linear gradient of 5-100% CH_3_CN over 20 min, and the flow rate was set to 70 mL/min. The collected fractions were analyzed via LCMS in order to determine the purity. Finally, the purified fractions were lyophilized. Analyses of the CRAMP_18–35_ were performed by UPLC-MS on a QTOF Impact HD, RSLC Dionex UltiMate 3000 (Thermo, Waltham, MA, USA) system using a Kinetex 2.6 µm EVO 100 Å C18 column (50 × 2.1 mm) (Phenomenex, Torrance, CA, USA) with a flow rate of 0.5 mL/min. The following solvent system was used: solvent A, water containing 0.1% formic acid; solvent B, acetonitrile containing 0.1% formic acid. The column was eluted using a linear gradient from 5–100% of solvent B. HR-MS: calcd. for C_109_H_182_N_28_O_22_ 2235.3982; found [M + 2H]^2+^ = 1118.7069, and [M + 3H]^3+^ = 746.1405, and [M + 4H]^4+^ = 559.8568, and [M + 5H]^5+^ = 448.0874. ^1^H NMR (400 MHz, D_2_O) δ 7.21–7.30 (m, 6H of phenyl), 7.15 (d, *J* = 7.7 Hz, 2H of phenyl), 7.06 (d, *J* = 7.2 Hz, 2H of phenyl), 4.55 (t, *J* = 6.9 Hz, 2H), 4.41–4.48 (m, 2H), 4.38 (d, *J* = 8.0 Hz, 1H), 4.31–4.09 (m, 13H), 4.06 (d, *J* = 8.2 Hz, 1H), 3.94–3.73 (m, 4H), 3.66–3.58 (m, 3H), 3.04–2.99 (m, 1H), 2.91–2.82 (m, 15H), 2.65–2.55 (m, 2H), 2.44 (s, 2H), 2.32 (s, 1H alkyne CH), 2.28–2.17 (m, 5H), 2.08–1.80 (m, 10H), 1.77–1.05 (m, 50H methylene protons of Lys, Leu, Ile, Pro), 0.90–0.75 (m, 30H Methyl protons of Leu, Ile, Val).

#### 3.1.2. Preparation of Chitosan Azide (**A1**)

Chitosan (250 mg, 1.479 mmol) was dissolved in 25 mL of 0.1 M HCl solution, then NaHCO_3_ (124 mg, 1.0 equiv.) was added to the solution, and the mixture was stirred vigorously for 30 min. After that, imidazole sulfonyl azide hydrochloride (124 mg, 0.591 mmol, 0.4 equiv.) and NaHCO_3_ (620 mg, 5.0 equiv.) were added slowly in small portions. Then, a solution of CuSO_4_·5H_2_O (48 mg, 0.192 mmol) in 1 mL of water and 5 mL of methanol solution was added to the reaction mixture. The reaction mixture turned bluish and was stirred further at room temperature for 24 h. Finally, the material was precipitated using acetone. The precipitate was filtered and washed with water, then acetone. The product was dried. Yield: 190 mg. A sharp azide peak was confirmed by IR spectroscopy at 2113 cm^−1^.

#### 3.1.3. Synthesis of Chitosan–CRAMP_18–35_ Peptide Conjugation via CuAAC Reaction (**1**)

Chitosan azide (10 mg, 0.0534 mmol) was dissolved in DMSO (2 mL) at 50 °C in the sealed tube. Then, CuSO_4_ 5H_2_O (0.13 equiv. in 0.5 mL water) and sodium ascorbate (0.5 equiv. in 0.5 mL water) were added, then pentynoyl–CRAMP_18–35_ peptide (119 mg, 0.0534 mmol) was added to the reaction mixture followed by THPTA ligand (23 mg, 0.0534 mmol). The reaction mixture was stirred at 50 °C for 48 h. Then, the resulting material was dialyzed against water for four days (first day 5% NaCl, following three days water), and freeze-dried. Yield: 30 mg. ^1^H NMR (400 MHz, D_2_O): δ 7.92 (triazole CH), 7.32–7.03 (peptide phenyl protons 3H), 4.63–4.04 (H2, H3, triazole CH_2_, and peptide protons 6H), 3.92–3.32 (H4, H5, H6, and peptide protons 7H), 3.05–2.83 (H6′, and peptide protons 5H), 2.69–2.55 (peptide protons 2H), 2.31–2.17 (peptide protons 2H), 2.09–1.10 (CS-(N-COCH_3_), and peptide protons 17H), 0.94–0.73 (peptide protons 7H).

#### 3.1.4. Synthesis of N-Hydroxypropyl Chitosan (HPC) (**A2**)

Chitosan (0.5 g) was basified by adding 10 mL of 30% NaOH aqueous solution and stirred at room temperature for 2 h. Then, the reaction mixture was kept at −18 °C for 24 h. After that, the mixture was thawed, isopropyl alcohol 15 mL was added and stirred at room temperature for 1 h, then propylene oxide (10 mL) was added with stirring for over 5 min. The suspension was further stirred at 45 °C for 16 h. The resulting product was neutralized by adding hydrochloric acid and dialyzed using a cellulose tube against de-ionized water for four days (first day 5% NaCl, following three days water) and freeze-dried to afford colorless HPC. Yield: 580 mg. ^1^H NMR (400 MHz, D_2_O): *δ* 4.75 (H1 merging with D_2_O peak), 2.82–4.00 (H2, H3, H4, H5, H6), 2.08 (N-COCH_3_), 1.16–1.21 (HP-CH_3_).

#### 3.1.5. Preparation of N-Hydroxypropyl Chitosan Azide (HPC-N_3_) (**A3**)

The HPC azide (100 mg, 0.591 mmol) was dissolved in 20 mL of 0.1 M HCl solution, NaHCO_3_ (45 mg, 1.0 equiv.) was added to the solution, and the mixture was stirred vigorously for 30 min. After that, imidazole sulfonyl azide hydrochloride (124 mg, 0.591 mmol) and NaHCO_3_ (452 mg, 10.0 equiv.) were added slowly in small portions. Then, a solution of CuSO_4_·5H_2_O (19 mg, 0.076 mmol) in 1 mL of water and 3 mL of methanol solution was added to the reaction mixture. The reaction mixture turned bluish and was stirred at room temperature for 24 h. Finally, the material was precipitated using acetone. The precipitate was filtered and washed with water, then acetone. The product was dried. Yield: 90 mg. The conversion was confirmed by a sharp azide peak that could be observed in the IR spectrum at 2113 cm^−1^.

#### 3.1.6. Synthesis of HPC-CRAMP_18–35_ Peptide Conjugation via CuAAC Reaction (**2**)

HPC azide (5 mg, 0.0267 mmol) was dissolved in DMSO (2 mL) at 50 °C in a sealed tube. Then, CuSO_4_·5H_2_O (0.13 equiv. in 0.5 mL water) and sodium ascorbate (0.5 equiv. in 0.5 mL water) were added, then pentynoyl–CRAMP_18–35_ peptide (59 mg, 0.0267 mmol) was added to the reaction mixture, followed by THPTA ligand (12 mg, 0.0267 mmol). The reaction mixture was stirred at 50 °C for 48 h. Then, the resulting material was dialyzed against water for four days (first day 5% NaCl, following three days water) and freeze-dried. Yield 17 mg. ^1^H NMR (400 MHz, D_2_O): δ 7.94 (triazole CH), 7.32–7.03 (peptide phenyl protons 1.4H), 4.62–4.01 (H2, H3, Triazole CH_2_, and peptide protons 4H), 3.96–3.22 (H4, H5, H6, and peptide protons 7H), 3.07–2.81 (H6′, and peptide protons 3.3H), 2.69–2.50 (peptide proton 2H), 2.34–2.17 (peptide protons 1H), 2.20–1.10 (CS-(N-COCH_3_), and peptide protons 9.2H), 1.18–0.98 (HP-CS proton 2.5H), 0.95–0.70 (peptide protons 5H).

#### 3.1.7. Synthesis of Chitotriazolan (**3**)

Chitosan azide (50 mg, 0.267) was dissolved in DMSO (5 mL) at 50 °C. Then, CuSO_4_·5H_2_O (0.13 equiv. in 1 mL water) and sodium ascorbate (0.5 equiv. in 1 mL water) were added to the reaction mixture, followed by *N*-propargyl-*N,N,N*-trimethylammonium bromide (1.5 equiv.) under nitrogen atmosphere. The reaction mixture was stirred at 50 °C for 48 h. Then, the resulting material was dialyzed against water for four days (first day 5% NaCl, following three days water) and freeze-dried. FT-IR spectra confirmed the disappearance of azide peak (at 2113 cm^−1^). Yield: 45 mg. ^1^H NMR (400 MHz, D_2_O): *δ* 8.59 (triazole CH), 5.19 (H1), 4.76 (triazole CH_2_ was merging with D_2_O peak), 4.60 (H2), 3.48–4.01 (H4, H5, H6), 4.47 (H3), 3.20 [*N*(CH_3_)_3_], 2.87 (H6′), 2.08 (N-COCH_3_),

#### 3.1.8. Synthesis of HP-Chitotriazolan (**4**)

HPC azide (50 mg, 0.267) was dissolved in DMSO (5 mL) at 50 °C. Then, CuSO_4_·5H_2_O (8 mg, 0.13 equiv. in 1 mL water) and sodium ascorbate (26 mg, 0.5 equiv. in 1 mL water) were added to the reaction mixture, followed by *N*-propargyl-*N,N,N*-trimethylammonium bromide (5 equiv.) under nitrogen atmosphere. The reaction mixture was stirred at 50 °C for 48 h. Then, the resulting material was dialyzed against water for four days (first day 5% NaCl, following three days water) and freeze-dried. FT-IR spectra confirmed the disappearance of azide peak (at 2113 cm^−1^). Yield 37 mg. ^1^H NMR (400 MHz, D_2_O): *δ* 8.58 (triazole CH_(tr)_), 5.22 (H1_(tr)_), 4.43–4.77 (triazole CH_2_, H3_(tr)_), 3.56–3.92 (H3-H6_(gh)_), H4-H6_(tr)_), 3.21 [*N*(CH_3_)_3(tr)_], 2.60–2.90 (H6′, H2_(gh)_), 2.08 (N-COCH_3_), 1.12–1.19 (HPC-CH_3(gh)_), The (gh) subscript identifies peaks assigned to the HPC glucosamine monomer.

### 3.2. Characterization

#### 3.2.1. NMR and FTIR Spectroscopy

The chitosan–peptide derivatives were characterized by using ^1^H NMR spectroscopy. ^1^H, COSY, and HSQC NMR spectra were recorded on a Bruker Avance 400 spectrophotometer operating at 400 MHz. NMR samples were prepared in D_2_O in 7–15 mg/mL concentrations. The N-acetyl peak (CH_3_CO) at 2.08 ppm was used as an internal reference in all proton NMR spectra. The degree of substitution was calculated based on NMR Spectroscopy. The chitosan (CS), chitosan azide, and chitotriazolan peptide derivatives were measured by FT-IR spectrometry using a Thermo Scientific™ Nicolet™ iS10 FTIR (from Roskilde, Denmark) spectrometer in the 500–4000 cm^−1^ wavelength region. The set number of scans was 64, and the resolution was 16 cm^−1^. A few milligrams of material were used to measure IR spectra, and all compounds were measured against a blank background.

#### 3.2.2. Gel Permeation Chromatography

Average molecular weight (Mw) and polydispersity index were determined via gel permeation chromatography (GPC) [40] using equipment from Polymer Standards Service (PSS) GmbH, Mainz, Germany, including the Agilent 1260 Infinity II GPC/SEC System, Isocratic Pump, GPC/SEC Column Thermostat, autosampler, Refractive Index Detector, and PSS’s ETA-2010 viscometer and MALLS detector (PPC SLD 7100). Data collection and processing were performed using WINGPC Unity 7.4 software. The GPC system comprised three columns: PSS Novema 10 µ guard (50 × 8 mm), PSS Novema 10 µ 30 Å (150 × 8 mm), and PSS Novema 10 µ 1000 Å (300 × 8 mm). Calibration was achieved using Ready Cal-Kit Pullulan standards with Mp (180–708,000 Da) from PSS GmbH, Mainz, Germany. A 0.1 M NaCl/0.1% TFA solution served as the eluent, with each sample dissolved in the same eluent at a 1 mg/mL concentration at 25 °C and a flow rate of 1 mL/min. Each sample was injected at a volume of 100 µL, with a 30 min interval between injections.

### 3.3. Antibacterial Assay for the Antimicrobial Chitosan Peptide Derivatives

Minimal inhibition concentration (MIC) and minimum lethal concentration (MLC) were measured according to the CLSI standard [50]. The antibacterial activity was tested against four different bacterial species: Gram-positive bacteria *S. aureus*, (ATCC 29213), and *E. faecalis*, (ATCC 29212), and Gram-negative bacteria *E. coli*, (ATCC 25922), and *P. aeruginosa*, (ATCC 27853). Before MIC testing, the bacterial strains were cultured on blood agar at 37 °C for 12–18 h. The bacterial colonies were suspended in saline water, adjusted to 0.5 McFarland, and further diluted in Mueller–Hinton broth (MHB) to reach the final concentration of 5 × 10^5^ colony-forming units (CFU)/mL in the test wells. The MHB was used for MIC measurement at pH 7.2. Gentamicin, a well-known antibiotic, was used as a performance control, MHB without chitosan derivatives or the bacterial solution as a sterility control, and MHB with only the bacterial solution as a growth control. The stock solution of compounds was prepared in sterile water at a concentration of 4096 µg/mL, then 50 µL of the compounds were added to a microtiter 96-well plate, and two-fold dilutions were done in MHB for concentrations 8–4096 µg/mL. Later, 50 µL of bacterial 5 × 10^5^ (CFU)/mL suspension was added to each well. The microtiter plates were incubated at 37 °C for 20 to 24 h. The naked eye observed the MIC values and determined the lowest concentrations of the antibacterial agent to completely inhibit the visible growth of microorganisms in the microtiter 96-well plate. For MLC measurement, the active antibacterial concentration of 10 μL × 2 of each dilution showed no visible growth and was plated on an agar plate and incubated at 35 °C for 20–24 h. MLC was defined as the lowest concentration that achieved a 99.9% decrease in viable cells.

## 4. Conclusions

The focus of the study was on preparing antimicrobial peptide chitosan conjugates by covalently grafting pentynoyl–CRAMP_18–35_ peptide onto chitosan through a CuAAC reaction. To ensure the success of the reaction, chitosan was synthesized to achieve a low degree of azidation, followed by the CuAAC reaction using pentynoyl–CRAMP_18–35_ peptide. The grafted chitosan–CRAMP_18–35_ peptide conjugates were characterized using proton NMR and IR spectroscopy techniques. The degree of substitution for chitosan–CRAMP_18–35_ peptide (**1**) and hydroxypropyl chitosan (HPC)-CRAMP_18–35_ peptide (**2**) was determined to be 0.2 and 0.13, respectively. This parameter indicates the extent to which the peptide is grafted onto the chitosan backbone, which was 10 to 20 times higher than was obtained with previously reported conjugation of Dhvar-5 peptide with the same type of linkage and click chemistry approach [44]. The antibacterial activity of chitosan–peptide conjugates was evaluated against four bacterial strains: *S. aureus*, *E. faecalis*, *E. coli*, and *P. aeruginosa*. Both chitosan–peptide conjugates **1** and **2** exhibited similar activity against all tested bacteria, with better activity observed only against the Gram-negative bacteria *E. coli* and *P. aeruginosa.* We are not aware of other investigations of antimicrobial chitosan conjugates where such remarkable selectivity has been reported. Conversely, the activity of other chitotriazolan (**3**) and hydroxypropyl chitotriazolan (HPC) (**4**) derivatives was reduced due to the low degree of substitution of quaternary trimethylated groups compared to our previous results [46]. HPC derivative (**A2**) activity remained consistent with previous results, indicating its reliability and potential for further applications [35]. These results suggest that chitosan–peptide conjugates hold promise for antibacterial applications. The future direction might be to apply these compounds in biomedical fields owing to their favorable antibacterial properties and potential versatility in various medical contexts, such as wound dressings, drug delivery systems, or antimicrobial coatings.

## Figures and Tables

**Table 1:** The DS, DA, and molecular weight for CRAMP_18–35_-chitotriazolan derivatives.

Derivatives	DS for Triazole CH	DS for Pep-phenyl	DS for Pep-Methyl	DS for HP-Methyl	DS for Triazole TM	DA	MW (Da)	Poly Dispersity Index (D)
**1**	0.20	0.23	0.22	NA	NA	ND	1.331 × 10^5^	1.08
**2**	0.13	0.12	0.12	0.65	NA	ND	2.931 × 10^5^	2.06
**3**	0.26	NA	NA	NA	0.34	0.11	4.138 × 10^4^	1.01
**4**	0.22	NA	NA	0.61	0.25	0.05	1.916 × 10^5^	3.59
**A2**	NA *	NA	NA	0.80	NA	0.06	1.959 × 10^5^	1.90

* NA—not available, ND—not detectable; DS—degree of substitution, calculated from NMR data, see materials and methods section; DA—degree of acetylation, calculated from NMR data; HP—hydroxypropyl.

**Table 2:** Antibacterial activity for CRAMP_18–35_-conjugated chitosan biopolymers (MIC and MLC).

		MIC Values in µg/mL			
Chitotriazolan Peptide Derivatives	Structures	*S. aureus* (ATCC 29213)	*E. faecalis* (ATCC 29212)	*E. coli* (ATCC 25922)	*P. aeruginosa* (ATCC 27853)
**1**	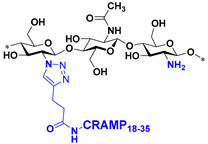	≥4096 *	≥4096 *	256	1024
**2**	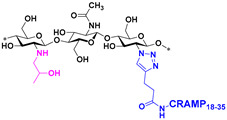	≥4096 *	≥4096 *	256	512
**3**	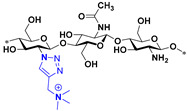	1024	4096	4096	4096
**4**	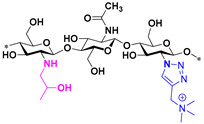	4096	4096	2048	4096
**A2**	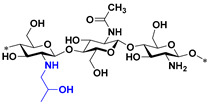	4096	4096	4096	4096
**A**	Pentynoyl–CRAMP_18–35_	128	128	8	64

* The availability of the peptide conjugates was more limited; in this case, the starting concentration was 2048 µg/mL.

## Data Availability

The data presented in this study is available in the article, as well as Supporting Information.

## Supplementary Materials

The following supporting information can be downloaded at: https://www.mdpi.com/article/10.3390/ijms25179440/s1.
