# Supplementary material for: Conjugation of CRAMP18–35 Peptide to Chitosan and Hydroxypropyl Chitosan via Copper-Catalyzed Azide–Alkyne Cycloaddition and Investigation of Antibacterial Activity
Source: Int J Mol Sci. 2024 Aug 30;25(17):9440. doi: 10.3390/ijms25179440 (PMC11394780; doi:10.3390/ijms25179440)
Supplement: Supplementary file 1 [file ijms-25-09440-s001.zip › ijms-3130607-supplementary.pdf]

Supporting Information for

Conjugation of $CRAMP_{18–35}$ Peptide to Chitosan and Hydroxypropyl Chitosan via Copper-Catalyzed Azide-Alkyne Cycloaddition and Investigation of Antibacterial Activity

Sankar Rathinam [1], Kasper K. Sørensen [2], Martha Á. Hjálmarsdóttir [3], Mikkel B. Thygesen [2], and Már Másson* [1]

[1] *Faculty of Pharmaceutical Sciences, School of Health Sciences, University of Iceland, Hofsvallagata 53, IS-107 Reykjavík, Iceland*; sankar@hi.is

[2] *Department of Chemistry, Faculty of Science, University of Copenhagen, Thorvaldsensvej 40, DK-1871 Frederiksberg C, Copenhagen, Denmark*; kso@chem.ku.dk (K.K.S.); mbth@chem.ku.dk (M.B.T.)

[3] *Department of Biomedical Science, Faculty of Medicine, School of Health Sciences, University of Iceland, Hringbraut 31, IS-101 Reykjavík, Iceland;* hjalmars@hi.is

Correspondence: mmasson@hi.is

The molecular weight distribution of the chitosan peptide conjugates, and the chitosan derivatives based on the GPC analysis.

Chitosan peptide Conjugates **1**

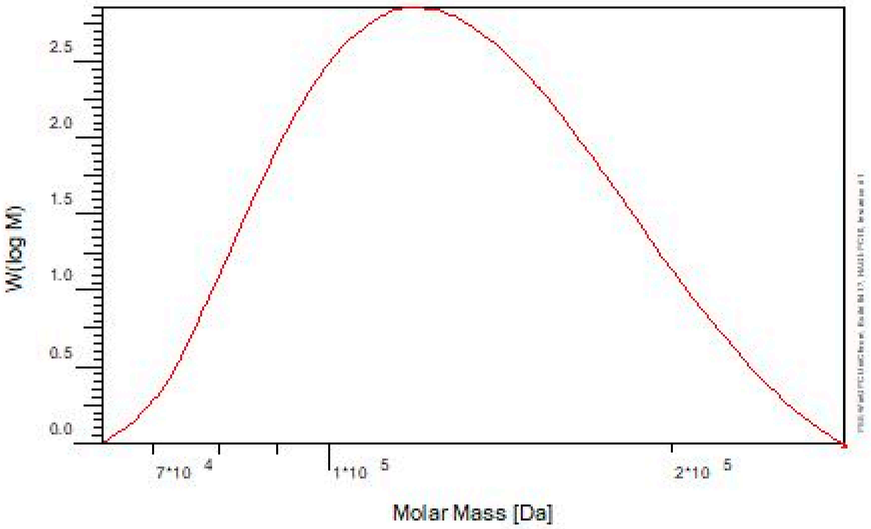

**Sample :**	Vial 12: RKS-6M-01 - 1		
**Integration from:**	Thursday 16.02.23 03:25:06		13.651 ml
**Integration to :**	Thursday 16.02.23 03:29:03		17.601 ml
**Calibration File :**	C:\Program Files (x86)\PSS WinGPC UniChrom[illegible]Calibration [illegible]uffer Nov 2022.CAL	**Eluent :**	[illegible]
**MHK - A (Cal.):**	0.000E+0	**MHK - K (Cal.):**	1.000E+0 ml/g
**Int.stand.-cal.:**	50.000 ml	**Int.stand.-sam.:**	------ ml
**Pump :**	n.a.	**Flowrate :**	1.000 ml/min
**Concentration :**	1.000 g/l	**Inject volume :**	100.000 ul
**Column 1 :**	PSS Novema 10µm precolumn	**Temperature :**	40.000 °C
**Column 2 :**	PSS Novema 10µm 30A	**Temperature :**	40.000 °C
**Column 3 :**	PSS Novema 10µm 1000A	**Temperature :**	40.000 °C
**Detector 1 :**	I1: RID 1, RI Signal	**Delay volume :**	0.583 ml
**Detector 2 :**	I1: IsoPump 1, Pressure	**Delay volume :**	0.000 ml
**Detector 3 :**	PSS DVD1260 IP	**Delay volume :**	0.299 ml
**Detector 4 :**	PSS DVD1260 DP	**Delay volume :**	0.299 ml
**Detector 5 :**	PSS SLD 7000/BI-MwA	**Delay volume :**	0.000 ml
**Operator :**	gpcadmin	**Acquisition interval**	1.000 sec

I1: RID 1, RI Signal

		Uncert.[%]	
**Mn :**	1.2234e5	n/a	g/mol
**Mw :**	1.3313e5	n/a	g/mol
**Mz :**	1.4518e5	n/a	g/mol
**Mv :**	0.000000	n/a	g/mol
**D :**	1.0882e0	n/a	
**[n]:**	0.000000	n/a	ml/g
**Vp :**	1.5918e1	256.75	ml
**Mp :**	1.1947e5	n/a	g/mol
**A :**	9.798e-4	256.75	ml*V
**< 63008**	0.00	256.75	
**w% :**	100.00	256.75	
**> 282982**	0.00	256.75	

Hydroxypropyl chitosan peptide Conjugates **2**

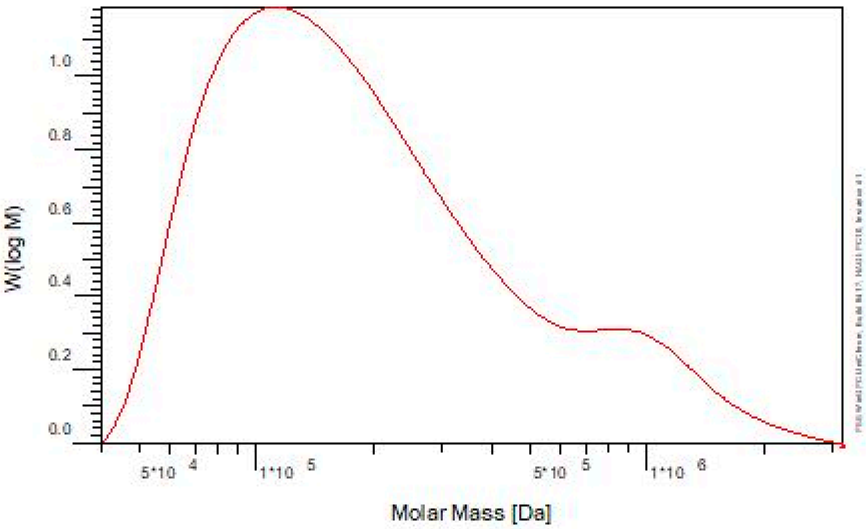

**Sample :**	Vial 13: RKS-6M-02 - 1		
**Integration from :**	Thursday 16.02.23 04:55:17		10.804 ml
**Integration to :**	Thursday 16.02.23 05:02:21		17.875 ml
**Calibration File :**	C:\Program Files (x86)\PSS WinGPC UniChrom[illegible]Calibration [illegible]uffer Nov 2022.CAL	**Eluent** [illegible]	
**MHK - A (Cal.):**	0.000E+0	**MHK - K (Cal.):**	1.000E+0 ml/g
**Int.stand.-cal.:**	50.000 ml	**Int.stand.-sam.:**	------ ml
**Pump :**	n.a.	**Flowrate :**	1.000 ml/min
**Concentration :**	1.400 g/l	**Inject volume :**	100.000 ul
**Column 1 :**	PSS Novema 10µm precolumn	**Temperature :**	40.000 °C
**Column 2 :**	PSS Novema 10µm 30A	**Temperature :**	40.000 °C
**Column 3 :**	PSS Novema 10µm 1000A	**Temperature :**	40.000 °C
**Detector 1 :**	I1: RID 1, RI Signal	**Delay volume :**	0.583 ml
**Detector 2 :**	I1: IsoPump 1, Pressure	**Delay volume :**	0.000 ml
**Detector 3 :**	PSS DVD1260 IP	**Delay volume :**	0.299 ml
**Detector 4 :**	PSS DVD1260 DP	**Delay volume :**	0.299 ml
**Detector 5 :**	PSS SLD 7000/BI-MwA	**Delay volume :**	0.000 ml
**Operator :**	gpcadmin	**Acquisition interval**	1.000 sec

I1: RID 1, RI Signal

		Uncert.[%]	
**Mn :**	1.4198e5	n/a	g/mol
**Mw :**	2.9316e5	n/a	g/mol
**Mz :**	7.0027e5	130.66	g/mol
**Mv :**	0.000000	n/a	g/mol
**D :**	2.0649e0	n/a	
**[n]:**	0.000000	n/a	ml/g
**Vp :**	1.6195e1	17.45	ml
**Mp :**	1.1266e5	n/a	g/mol
**A :**	6.447e-3	17.45	ml*V
**< 39893**	0.00	17.45	
**w% :**	100.00	17.45	
**> 3158248**	0.00	17.45	

Chitotriazolan derivative **3**

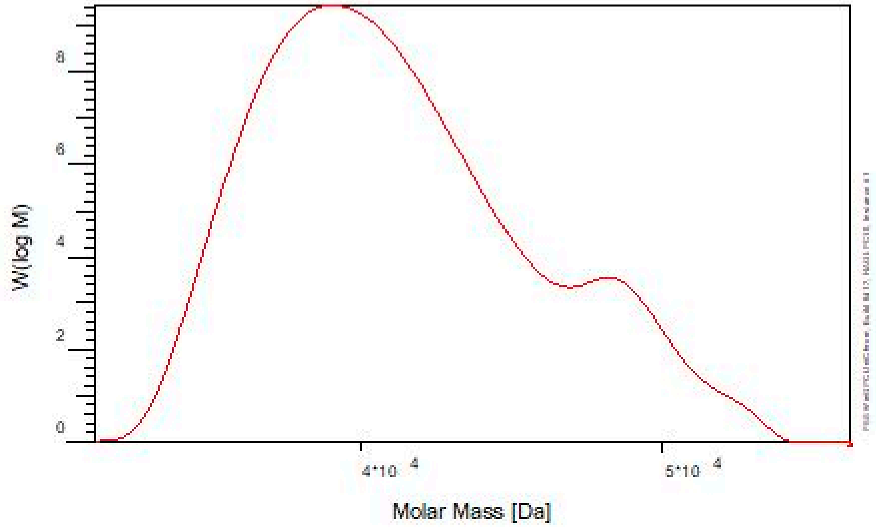

**Sample :**	Vial 14: RKS-6M-03 - 1		
**Integration from:**	Thursday 16.02.23 06:27:14		9.744 ml
**Integration to :**	Thursday 16.02.23 06:36:33		19.051 ml
**Calibration File :**	C:\Program Files (x86)\PSS WinGPC UniChrom[illegible]Calibration [illegible]uffer Nov 2022.CAL	**Eluent :**	[illegible]
**MHK - A (Cal.):**	0.000E+0	**MHK - K (Cal.):**	1.000E+0 ml/g
**Int.stand.-cal.:**	50.000 ml	**Int.stand.-sam.:**	------ ml
**Pump :**	n.a.	**Flowrate :**	1.000 ml/min
**Concentration :**	1.000 g/l	**Inject volume :**	100.000 ul
**Column 1 :**	PSS Novema 10µm precolumn	**Temperature :**	40.000 °C
**Column 2 :**	PSS Novema 10µm 30A	**Temperature :**	40.000 °C
**Column 3 :**	PSS Novema 10µm 1000A	**Temperature :**	40.000 °C
**Detector 1 :**	I1: RID 1, RI Signal	**Delay volume :**	0.583 ml
**Detector 2 :**	I1: IsoPump 1, Pressure	**Delay volume :**	0.000 ml
**Detector 3 :**	PSS DVD1260 IP	**Delay volume :**	0.299 ml
**Detector 4 :**	PSS DVD1260 DP	**Delay volume :**	0.299 ml
**Detector 5 :**	PSS SLD 7000/BI-MwA	**Delay volume :**	0.000 ml
**Operator :**	gpcadmin	**Acquisition interval**	1.000 sec

	**I1: RID 1, RI Signal**	**Uncert.[%]**	
**Mn :**	4.0970e4	n/a	g/mol
**Mw :**	4.1387e4	n/a	g/mol
**Mz :**	4.1828e4	n/a	g/mol
**Mv :**	0.000000	n/a	g/mol
**D :**	1.0102e0	n/a	
**[n]:**	0.000000	n/a	ml/g
**Vp :**	1.6074e1	7.38	ml
**Mp :**	3.9278e4	n/a	g/mol
**A :**	1.203e-2	7.38	ml*V
**< 32840**	0.00	7.38	
**w% :**	100.00	7.38	
**> 57470**	0.00	7.38	

HP-Chitotriazolan **4**

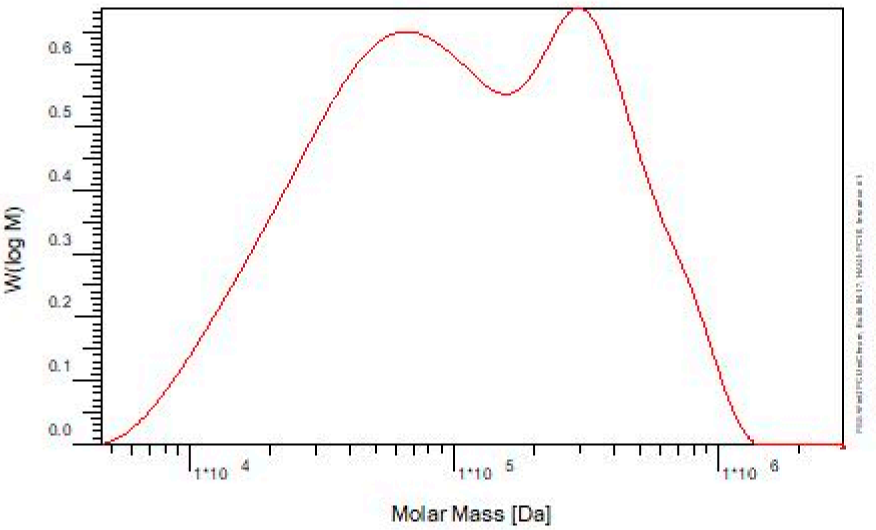

**Sample :**	Vial 15: RKS-6M-04 - 3		
**Integration from :**	Thursday 16.02.23 09:01:56		9.388 ml
**Integration to :**	Thursday 16.02.23 09:10:40		18.137 ml
**Calibration File :**	C:\Program Files (x86)\PSS WinGPC UniChrom\Client\Calibration V[illegible]uffer Nov 2022.CAL	**Eluent :**	[illegible]
**MHK - A (Cal.):**	0.000E+0	**MHK - K (Cal.):**	1.000E+0 ml/g
**Int.stand.-cal.:**	50.000 ml	**Int.stand.-sam.:**	------ ml
**Pump :**	n.a.	**Flowrate :**	1.000 ml/min
**Concentration :**	1.000 g/l	**Inject volume :**	100.000 ul
**Column 1 :**	PSS Novema 10µm precolumn	**Temperature :**	40.000 °C
**Column 2 :**	PSS Novema 10µm 30A	**Temperature :**	40.000 °C
**Column 3 :**	PSS Novema 10µm 1000A	**Temperature :**	40.000 °C
**Detector 1 :**	I1: RID 1, RI Signal	**Delay volume :**	0.583 ml
**Detector 2 :**	I1: IsoPump 1, Pressure	**Delay volume :**	0.000 ml
**Detector 3 :**	PSS DVD1260 IP	**Delay volume :**	0.299 ml
**Detector 4 :**	PSS DVD1260 DP	**Delay volume :**	0.299 ml
**Detector 5 :**	PSS SLD 7000/BI-MwA	**Delay volume :**	0.000 ml
**Operator :**	gpcadmin	**Acquisition interval**	1.000 sec

I1: RID 1, RI Signal

		Uncert.[%]	
**Mn :**	5.3320e4	n/a	g/mol
**Mw :**	1.9167e5	n/a	g/mol
**Mz :**	4.2164e5	n/a	g/mol
**Mv :**	0.000000	n/a	g/mol
**D :**	3.5948e0	n/a	
**[n]:**	0.000000	n/a	ml/g
**Vp :**	1.2499e1	9.07	ml
**Mp :**	2.9352e5	n/a	g/mol
**A :**	9.143e-3	9.07	ml*V
**< 4578**	0.00	9.07	
**w% :**	100.00	9.07	
**> 2914345**	0.00	9.07	

Hydroxypropyl chitosan **A2**

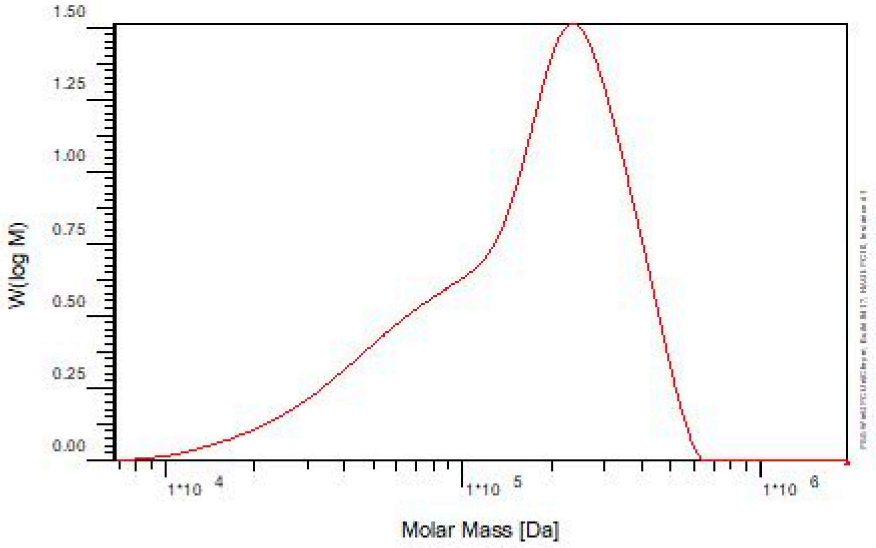

**Sample :**	Vial 16: RKS-6M-05 - 2		
**Integration from:**	Thursday 16.02.23 10:03:21		8.787 ml
**Integration to :**	Thursday 16.02.23 10:12:36		18.037 ml
**Calibration File :**	C:\Program Files (x86)\PSS WinGPC UniChrom[illegible]Calibration [illegible]uffer Nov 2022.CAL	**Eluent :**	[illegible]
**MHK - A (Cal.):**	0.000E+0	**MHK - K (Cal.):**	1.000E+0 ml/g
**Int.stand.-cal.:**	50.000 ml	**Int.stand.-sam.:**	------ ml
**Pump :**	n.a.	**Flowrate :**	1.000 ml/min
**Concentration :**	1.000 g/l	**Inject volume :**	100.000 ul
**Column 1 :**	PSS Novema 10µm precolumn	**Temperature :**	40.000 °C
**Column 2 :**	PSS Novema 10µm 30A	**Temperature :**	40.000 °C
**Column 3 :**	PSS Novema 10µm 1000A	**Temperature :**	40.000 °C
**Detector 1 :**	I1: RID 1, RI Signal	**Delay volume :**	0.583 ml
**Detector 2 :**	I1: IsoPump 1, Pressure	**Delay volume :**	0.000 ml
**Detector 3 :**	PSS DVD1260 IP	**Delay volume :**	0.299 ml
**Detector 4 :**	PSS DVD1260 DP	**Delay volume :**	0.299 ml
**Detector 5 :**	PSS SLD 7000/BI-MwA	**Delay volume :**	0.000 ml
**Operator :**	gpcadmin	**Acquisition interval**	1.000 sec

	I1: RID 1, RI Signal	Uncert.[%]	
**Mn :**	1.0266e5	36.98	g/mol
**Mw :**	1.9590e5	24.75	g/mol
**Mz :**	2.7195e5	6.55	g/mol
**Mv :**	0.000000	27.45	g/mol
**D :**	1.9082e0	44.49	
**[n]:**	0.000000	0.00	ml/g
**Vp :**	1.2233e1	2.90	ml
**Mp :**	2.3575e5	37.42	g/mol
**A :**	1.041e-2	2.90	ml*V
**< 6742**	0.00	2.90	
**w% :**	100.00	2.90	
**> 1945883**	0.00	2.90	
